# Preliminary experience with recanalization of large vessel occlusion due to underlying long-segment dissection using a standby microwire technique

**DOI:** 10.3389/fneur.2022.1016734

**Published:** 2022-11-30

**Authors:** Xiaoxi Zhang, Hongye Xu, Zhengzhe Feng, Weilong Hua, Hongjian Shen, Lei Zhang, Yongxin Zhang, Pengfei Xing, Pengfei Yang, Xiaolong Xu, Zifu Li

**Affiliations:** Neurovascular Center, Changhai Hospital, Naval Medical University, Shanghai, China

**Keywords:** stroke, thrombectomy, technique, dissection, experience

## Abstract

**Purpose:**

This study aimed at investigating a novel standby microwire technique to facilitate revascularization of large vessel occlusion due to underlying long-segment dissection.

**Methods:**

Patients with acute ischemic stroke with emergent large vessel occlusion (ELVO) due to underlying long-segment dissection were screened from the prospectively established database between January 2021 and May 2022. The clinical and radiological data of eligible patients who underwent endovascular treatment by using a standby microwire technique were investigated.

**Results:**

Of the 165 acute ischemic stroke patients who underwent mechanical thrombectomy, the standby microwire technique was used in five patients aged 33–55 years old with occlusion due to underlying long-segment dissection. Of them, three patients were diagnosed with tandem lesions and three were located at the anterior circulation. A 300 cm exchange microwire was used as the standby microwire. Stent deployment was performed in all five patients. Groin puncture to reperfusion time ranged from 10–68 min. Technical success and favorable clinical outcomes were achieved in all five patients (100%). No technique-related complication was observed.

**Conclusion:**

Our preliminary experience showed that the standby microwire technique was a useful ancillary approach to facilitate the revascularization of large vessel occlusion due to underlying long-segment dissection.

## Introduction

Mechanical thrombectomy (MT) has been accepted as first-line treatment for emergent large vessel occlusion (ELVO) due to internal carotid artery (ICA) or vertebral artery (VA) dissection as an infrequent entity. However, it may potentially lead to severe and catastrophic acute ischemic stroke (AIS) (1). Vascular dissection accounts for ~2% of AIS in the general population (2) and 10–25% in patients under the age of 50 years (3). MT for LVO caused by dissection remains a clinical challenge (4). One of the reasons is that ICA or vertebral dissection is commonly accompanied with long-segment vessel injury and massive thrombus burden, making it difficult to be revascularized in a short time. In addition, intracranial mechanical thrombectomy is difficult and time consuming due to difficult access and the need for revascularization of the long and complex lesions (1).

The subsequent development of the technique of preserving the true lumen for repeated thrombectomy makes the procedure safer and less time-saving. In this article, we present a novel technique of “standby microwire technique” using an exchange microwire to ensure the patency of the dissection lumen during the endovascular procedure.

## Methods

Patients diagnosed with AIS due to long-segment dissection who underwent endovascular treatment were enrolled from Jan 1, 2021 to May 30, 2022. Clinical and radiological data were obtained from the prospectively collected database. Written and signed informed consent was obtained from all participating patients.

Long-segment dissection was defined as dissection extending over at least two artery segments on the first angiogram. The study was approved by the institutional review board. According to local regulations, patient consent was not required for this study which involved strictly anonymized data. The data supporting the findings of the present study were available from the corresponding author on reasonable request. The following procedural outcomes were prospectively assessed. Successful recanalization was defined as extended Thrombolysis in Cerebral Infarction (eTICI) 2b/3. Technical success was defined as eTICI 2c/3 after using the standby microwire technique without the use of rescue therapy. Procedure-associated complications included arterial perforation, new arterial dissection, embolization in a new territory, or subarachnoid hemorrhage. Technique-related complications included tanglement of the devices, difficulty with stent retrieval, and arterial perforation due to the exchange microwire and bounce of the exchange microwire. The favorable clinical outcome rate was defined as a modified Rankin Scale (mRS) score at 90 days of 0–2 or equal to pre-stroke mRS. Symptomatic intracranial hemorrhage was defined as any intracranial hemorrhage visualized in follow-up imaging study and associated with a worsening of 4 or more points on the NIHSS score or death.

## Technique details

**Figure 1:**
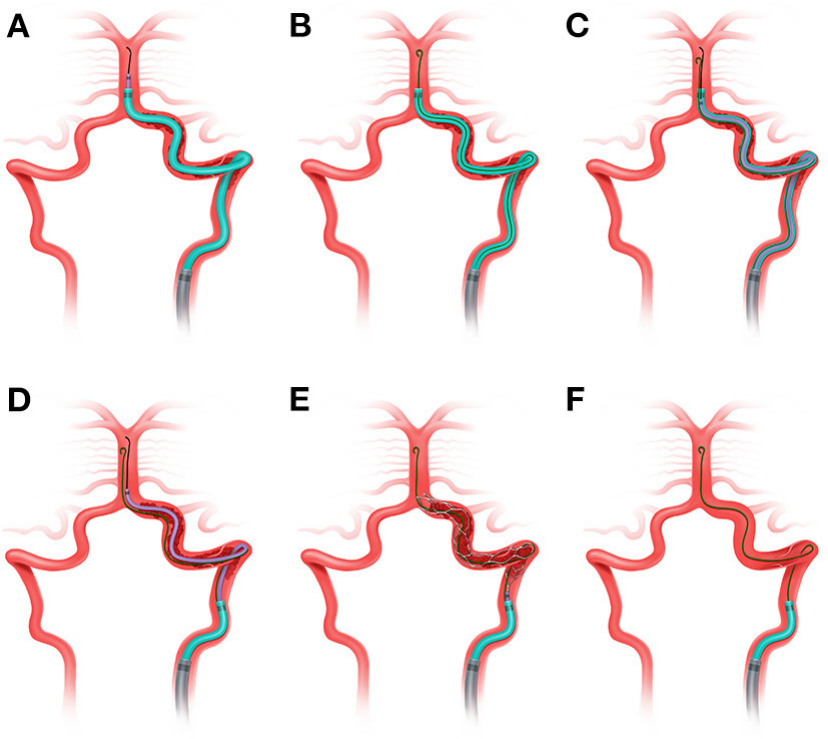
Illustration of “standby microwire technique”. **(A)** The guiding catheter (gray) was advanced to V1 segment, the aspiration catheter (light green) and microcatheter (purple) were advanced to the basilar artery with the assistance of Synchro microwire (black). **(B)** A Floppy 300 microwire (dark green) was then exchanged with Synchro microwire and stayed in the basilar artery. The tip was shaped into protective “J” (black arrow). **(C)** The microcatheter was then advanced through the dissection to the basilar artery. **(D)** The aspiration catheter was retrieved to proximal part of dissection under continuous aspiration. **(E)** Synchro microwire was withdrawn and the self-expandable stent retriever was advanced to the distal part of dissection and was then delivered. **(F)** The thrombus was removed with the stent through the aspiration catheter, without affecting the position of the exchange microwire.

The key step of standby microwire technique is illustrated in Figure 1. Three key steps are as follows.

### Super-selection of true lumen

Intermediate catheter (5F or 6F Catalyst, Stryker, USA) was advanced with the tri-axial technique, afterward, a microwire (synchro 0.014/150, Stryker, USA) supported by the microcatheter was manipulated gently to cross the occluded segment. If needed, contralateral access was established to provide the roadmap of the distal lumen, especially for VA occlusion with underlying long-segment dissection. After passing through the dissection, angiography was performed *via* the microcatheter to verify the entrance of the true lumen. Then, an intermediate catheter was advanced to the distal normal lumen.

### Preservation of true lumen with exchange microwire

Afterward, the tip of exchange microwire, with 300 cm length and 0.014 inch diameter, would be shaped into a “J” shape and stayed in the true lumen. After retrieval of the microcatheter, the microcatheter supported by the microwire would be re-advanced through the intermediate catheter to perform mechanical thrombectomy. When the stent retriever was in position, the intermediate catheter was pulled back to the proximal normal lumen.

### Thrombectomy

Then, thrombectomy was performed. If second thrombectomy needed, the intermediate catheter was re-advanced to the distal normal lumen along with the standby microwire. Afterwards, angioplasty with balloon dilation or stent implantation could be performed with the assistance of the standby microwire if necessary. Unnecessary angiography should be avoided to reduce the risk of thrombus migration. The whole system was retrieved after successful revascularization. Tirofiban was initiated during MT if stenting was performed, and maintained for 36 h. Thrombo-elastography with platelet mapping (TEG) was performed after MT.

## Results

Of the five patients included in the analysis, the mean age was 46.2 ± 8.9 years, the median NIHSS score was 9 (5–20), the median initial NCCT ASPECTS or pc-ASPECTS (5) was 9 (6–10), and the median time from symptom onset to revascularization was 277 (123–795) min (Table 1). Two patients had tandem lesions, with proximal occlusion of the ICA and distal occlusion of the MCA. The successful reperfusion status, defined as Thrombolysis In Cerebral Infarction (TICI) 2b-3, was achieved in all 5 cases (Table 1).

**Table 1:** Characteristics of enrolled patients.

	**Age/sex**	**Medical history**	**Occlusion site**	**NIHSS**	**ASPECTS**	**Thrombolysis**	**General anesthesia**
Case 1	33/M	Neck massage	L-VA	9	10	N	Y
Case 2	44/M	/	Tandem R-ICA & R-M1	9	10	Y	N
Case 3	55/M	DM/HTN	R-ICA	20	7	Y	Y
Case 4	45/M	DM	Tandem L-ICA & L-M2	5	9	Y	N
Case 5	54/M	/	L-VA	9	6	N	Y
	**Exchange microwire**	**SR/AC/AP**	**Stent implanted**	**TICI**	**Complications**	**Time from onset to recanalization**	**Prior mRS**	**m mRS**
Case 1	Floppy 300	2/1/3	Enterprise/Leo	3	None	4 h 37 min	0	0
Case 2	Floppy 300	1/0/1	Wallstent	3	None	2 h 56 min	0	0
Case 3	Synchro 300	0/0/1	Xpert	3	None	10 h 35 min	0	1
Case 4	Floppy 300	0/1/1	Xpert	3	None	2 h 3 min	0	0
Case 5	Floppy 300	2/3/2	Enterprise*2	2b	None	13 h 15 min	0	3

ASPECTS: Alberta stroke program early CT score; DM: diabetes mellitus; HTN: hypertension; ICA: internal carotid artery; NIHSS: National Institute of Health stroke scale; VA: vertebral artery. SR/AC/AP: times of stent retriever, aspiration and angioplasty used. Complications included hemorrhagic complications including perforator bleeding, intracranial hemorrhage, subarachnoid hemorrhage, puncture hematoma and ischemic complications including new territory stroke, perforator occlusion, re-occlusion and intra-stent occlusion.

Thrombolysis was performed in three patients prior to endovascular treatment. Endovascular treatment was performed under conscious sedation in two patients, and under general anesthesia in the other three patients. Thrombectomy was performed even in case of low NIHSS, knowing that symptom fluctuation in this patient was due to involvement of a very eloquent area. A floppy microwire and a synchro 0.014 inch microwire with length of 300 cm were used as the exchange microwire during the procedures. Extra stent implantation was required in all patients (Table 1). No bounce or tanglement was observed. Four out of the five patients resulted in a favorable clinical outcome in terms of modified Rankin Scale (mRS) ≤ 2 at 3 months. There were neither intra- nor peri-interventional complications.

### Illustrative case

A 33-year-old male patient complained of sudden aphasia and numbness of the left extremities for 5 h. The patient complaint of neck pain upon neck massage, which progressively extended from the neck to occipital region, face and left extremities. He also reported aphasia, choking and even loss of consciousness on one occasion. The patient was suspected with cerebral infarction in a local hospital, but ineligible for thrombolysis. Then, he was quickly transferred to our hospital. At admission, the NIHSS score was 9. CT showed no hypo-intensity or brain edema. CT perfusion showed penumbra volume = 5 ml. No special previous medical and disease history was recorded.

**Figure 2:**
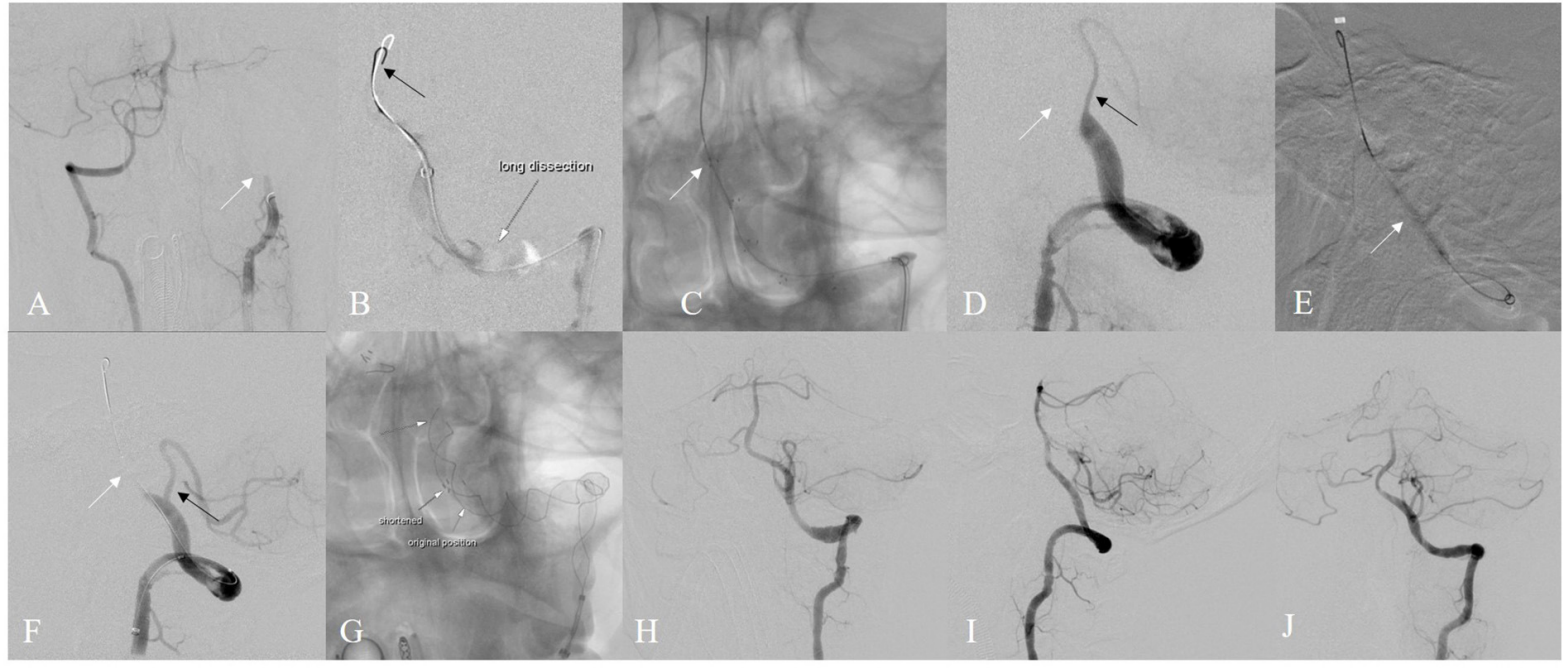
Illustrative case of “standby microwire technique” **(A)** left VA was occluded **(B)** superselection of exchange catheter through long segmental dissection **(C)** “J” sahpe microwire remained in the dissection **(D)** after 1st thrombectomy **(E)** balloon dilation was then used **(F)** partial recanalization **(G)** stent implantation was performed **(H,I)** eTICI 3 grade recanalization was achieved **(J)** excellent recovery after 6 months.

Mechanical thrombectomy was performed under general anesthesia (Figure 2). The femoral route was established with a long sheath (Infinity, Stryker, USA). Afterward, a thorough angiography was performed to evaluate the collaterals and dissection profiles. The first angiogram showed left VA occlusion (Figure 2A arrow), potentially caused by dissection; a synchro microwire was super-selected to the basilar tip after several attempts. Gentle angiography from the guiding catheter showed a long segmental dissection in the left VA (Figure 2B) and then the 300 cm microwire (black arrow) was shaped into a “J” and exchanged with the synchro microwire as a standby microwire. The 300cm microwire remained at the basilar tip (Figure 2C). Meanwhile, a Solitaire platinum stent (4 × 40 mm) was delivered to the dissection and expanded. After first thrombectomy with the stent retriever, the anterior inferior cerebellar artery was not recanalized and intermediate catheter was repositioned at the distal true lune along the standby microwire. Then microcatheter supported by microwire was advanced to distal true lumen and second thrombectomy was performed and PICA (black arrow) could be partially observed (Figure 2D). Balloon was advanced to the dissection segment along the standby microwire and dilation was subsequently performed (Figure 2E). Blood flow of the VA (white arrow) and PICA was further improved (Figure 2F). An Enterprise 2 stent (4 × 39 mm) was then implanted, followed by a Leo stent (4.5 × 75 mm) (Figure 2G). Finally, eTICI 3 grade recanalization was achieved (Figures 2H,I). Six months later, angiography showed excellent recovery of the VA and PICA (Figure 2J).

## Discussion

In this study, we reported recanalization of large vessel occlusion due to underlying long-segment dissection using a standby microwire technique in five cases. In this technique involves the use of an exchange microwire in performing mechanical thrombectomy or angioplasty. The standby microwire embedded in the dissection was used to preserve the lumen of dissection. The technique was successfully performed in all cases, without technique-related complications or affecting thrombectomy and angioplasty.

Dissection is an infrequent, albeit catastrophic form of stroke in the young (6). Given the long-segment and complex injury of the ICA and VA, identification of the true lumen is quite difficult in endovascular treatment (1). In clinical practice, the true lumen is commonly identified after several attempts. Thereafter, preservation of the true lumen is quite essential for the following steps (7). Our experience demonstrates that the standby microwire technique can provide a potential method for stable and safe preservation of the true lumen. In addition, the standby microwire does not interfere with the thrombectomy and angioplasty processes.

There are several challenges in managing dissection of ICA and VA (8). First, identification of the true lumen is the priority but most difficult step before performing the subsequent treatment steps. Before identification of the true lumen, angiography should be avoided to prevent the enlargement of the false lumen and extrusion of the true lumen, leading to failure of super-selection. Once proceeding the catheter and microwire through the false lumen, the true lumen might be thinner by rude manipulation. This is a one-way door decision, namely we might only have one chance to pass through the true lumen. Thereafter, the preservation of the true lumen is the key step. Second, after thrombectomy with the stent retriever or aspiration, the thrombectomy system including the microcatheter and stent was totally retrieved from the dissected lumen. If not successfully revascularized, the true lumen would disappear and become more difficult to pass for the second time than the first. Thirdly, dissection is commonly complex and involves a long segmental lesion, leading to long puncture-to-revascularization time. If thrombectomy fails, balloon dilation and stent implantation are alternative techniques. A standardized anti-platelet regimen is required.

Spontaneous cervical artery dissection has been associated with violent, sudden neck movements including neck massage and golf (9). A young man who complained of neck pain, blurred speech and limb numbness during neck massage was diagnosed with left VA dissection (Figure 2). In this case, the true lumen was identified after over 20 min of attempt. The large internal lumen and smooth drug-eluting inner membrane of guiding catheter facilitates the conduction of standby microwire technique during thrombectomy in this patient.

Take-home message of the technique is as follows. First, the tip of the microwire should always stay on the screen and the tension should be noted during the procedure, preventing potential perforator bleeding by bouncing of the microwire. Second, tangling of the exchange microwire and stent retriever should be noted by the standardized procedure protocol. Also, this study has some limitations. First, the sample size was small due to rare occurrence of long-segment dissection. In addition, statistical analysis was not performed due to the small sample size.

## Conclusion

We described a modified standby microwire technique assisted by a long exchange microwire revascularize large vessel occlusion due to underlying long-segment dissection, with satisfactory clinical outcomes. As our experience is preliminary and the sample size is relatively small, further studies with larger series are required to evaluate the safety and efficacy of this technique for the treatment of emergent large vessel occlusion with underlying long-segment dissection.

## Data availability statement

The raw data supporting the conclusions of this article will be made available by the authors, without undue reservation.

## Ethics statement

The studies involving human participants were reviewed and approved by Changhai Ethics Committee. The patients/participants provided their written informed consent to participate in this study.

## Author contributions

All authors listed have made a substantial, direct, and intellectual contribution to the work and approved it for publication.

## Funding

This work was funded by Shanghai Sailing Program No.: 20YF1448000.

## Conflict of interest

The authors declare that the research was conducted in the absence of any commercial or financial relationships that could be construed as a potential conflict of interest.

## Publisher's note

All claims expressed in this article are solely those of the authors and do not necessarily represent those of their affiliated organizations, or those of the publisher, the editors and the reviewers. Any product that may be evaluated in this article, or claim that may be made by its manufacturer, is not guaranteed or endorsed by the publisher.
